# Is your lunch salad safe to eat? Occurrence of bacterial pathogens and potential for pathogen growth in pre-packed ready-to-eat mixed-ingredient salads

**DOI:** 10.1080/20008686.2017.1407216

**Published:** 2017-12-01

**Authors:** Karin Söderqvist

**Affiliations:** ^a^ Department of Biomedical Sciences and Veterinary Public Health, Swedish University of Agricultural Sciences, Uppsala, Sweden

**Keywords:** Deli salad, *Listeria monocytogenes*, pathogenic *Yersinia enterocolitica*, *Salmonella enterica*, shiga toxin-producing *Escherichia coli*, temperature abuse

## Abstract

As part of a trend toward healthy convenience foods, ready-to-eat (RTE) mixed-ingredient salads have become popular products among consumers. A mixed-ingredient salad contains combinations of raw (*e.g*. leafy vegetables and tomatoes) and processed (*e.g*. chicken, salmon, ham, pasta and couscous) ingredients. Contamination of leafy vegetables can occur during any step in the production chain and, since there is no step that kills pathogens, a completely safe final product can never be guaranteed. Meat ingredients, for example poultry meat and ham, are generally heat-treated before preparation, but may be contaminated after this treatment, *e.g*. when diced or sliced. When several ingredients are mixed together, cross-contamination may occur. Preparation of mixed-ingredient salads requires human handling, which presents an additional risk of bacterial contamination. With high-protein ingredients, *e.g*. cooked meat, the mixed-ingredient salad represents an excellent substrate for bacterial growth. This article reviews current knowledge regarding human bacterial pathogen prevalence in mixed-ingredient salads and the potential for pathogen growth in this product during storage.

In recent years, there has been a development whereby different ingredients are added to vegetables to produce a wide range of salad meals for consumers [1]. Different terms have been used to describe this food product. ‘Pre-packaged mixed vegetable salad’ has been used by some [2,3], while ‘mixed-ingredient salad’ has been used by others [4,5] and is used in this review. The term ‘deli salad’ may be synonymous with ‘mixed-ingredient salad’ but may also refer to salads based on mayonnaise [6,7], which are not considered here. The definition of a ‘mixed-ingredient salad’ in this review is a ready-to-eat (RTE) product containing ingredients of both animal and non-animal origin. These salads are usually prepared by a manufacturer, but may also be prepared in-store, and are often stored for a few days before consumption. They are typically packaged in plastic bowls, readily available in the chill cabinets of supermarkets, cafés and convenience stores and intended as whole meals (Figure 1). A salad dressing is sometimes provided, but in a separate package intended to be added at the time of consumption, in order to avoid impairing the fresh appearance and crispy texture of the raw vegetables [8]. Mixed-ingredient salads that are prepared in salad bars based on customer preferences, and usually consumed instantly, are not addressed in this review.

**Figure 1.**
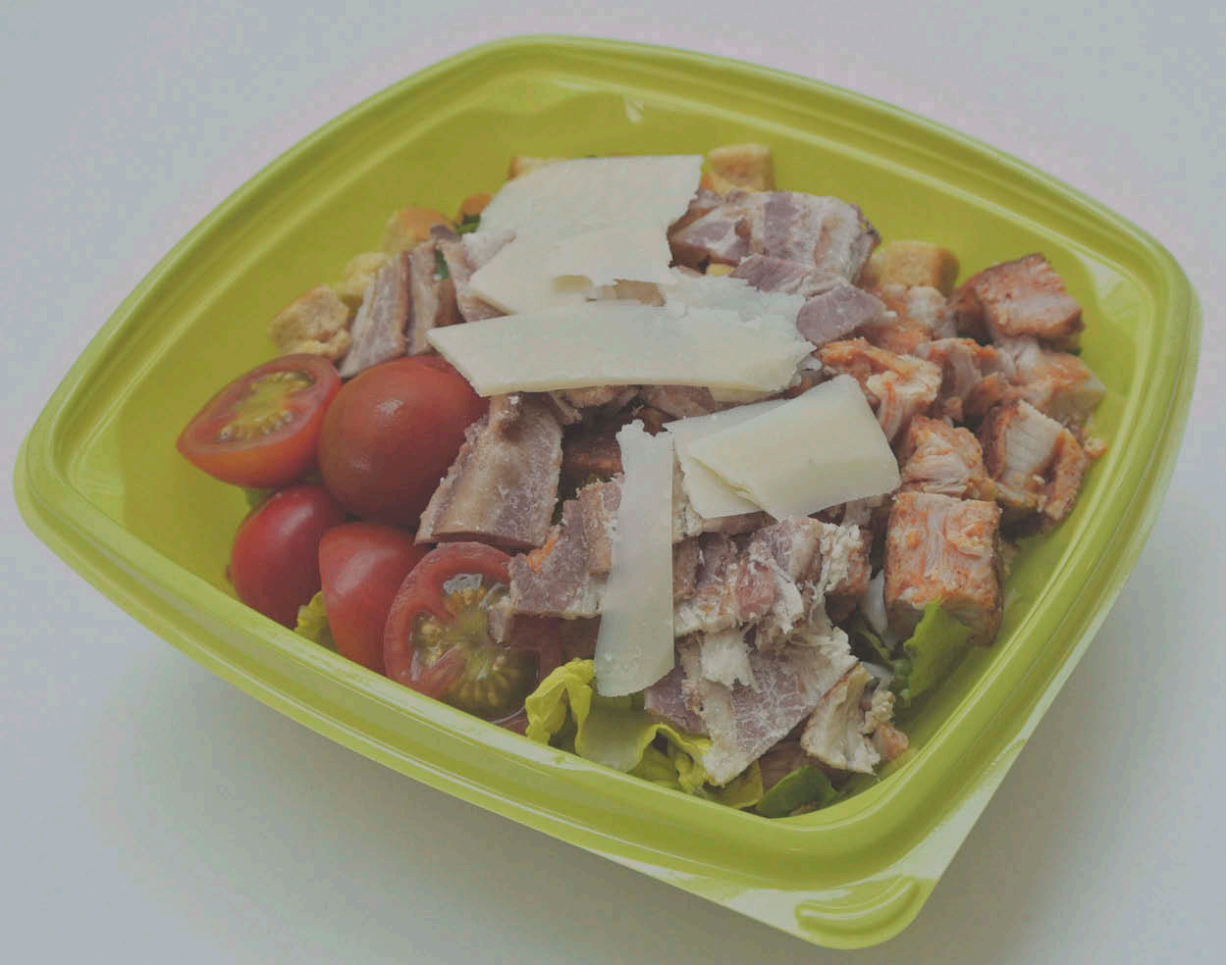
Commercial mixed-ingredient salad containing leafy vegetables, tomatoes, chicken, bacon and cheese.

To date, foods of animal origin have been the main source of documented and reported outbreaks of foodborne disease and 90% of the outbreaks in the European Union (EU) from 2007 to 2011 were associated with foods of animal origin [9]. However, the number of outbreaks and human cases associated with food of non-animal origin appears to be increasing [9]. In a risk assessment by the Food and Agriculture Organization of the United Nations (FAO), leafy vegetables were ranked the highest concern in terms of safety of fresh fruit and vegetables [10].

There are many foodborne pathogens that are relevant for RTE leafy vegetables, including bacteria, viruses and parasites. Among the viruses, norovirus and Hepatitis A have been linked to leafy vegetables [9]. *Cryptosporidium* spp., *Giardia* spp. and *Toxoplasma gondii* are examples of protozoan parasites that have been associated with foodborne illness from consuming leafy vegetables [9]. Examples of bacterial pathogens that have been associated with leafy vegetables are *Salmonella*, shiga toxin-producing *Escherichia coli* (STEC), *Campylobacter* spp., pathogenic *Yersinia enterocolitica* and *Listeria monocytogenes* [9,11]. Bacterial pathogens will be in focus in this review and most of these pathogens are faecal contaminants from animal or human carriers, but *L. monocytogenes* is ubiquitous in nature, including soil and vegetation [12].

There is little information about outbreaks of foodborne pathogens associated with RTE mixed-ingredient salads, but there have been recalls because of detection of e.g. *Salmonella, E. coli* O157:H7 and *L. monocytogenes* during internal monitoring of these products by food companies [13–15].

## Contamination of leafy vegetable ingredients in mixed-ingredient salads

Contamination of leafy vegetables with bacterial pathogens can occur during any step in the production chain. In a field environment, grazing livestock, wild animals, birds and insects are natural inhabitants and, since these may carry human pathogens [16–21], contamination of leafy vegetables can never be completely avoided. Irrigation water has been suggested as the major source of enteric pathogen contamination in leafy vegetables [22]. Despite this, few countries have established mandatory guidelines or standards on the microbiological quality of water used for irrigation [23]. Other possible sources of contamination include field workers with poor hygiene and equipment used during harvest and transport to the processing plant [24]. Postharvest contamination may occur via cross-contamination during washing or poor hygiene practices. There is no step that kills any pathogen possibly present during the production and processing of RTE leafy vegetables and thus a completely safe final product may never be guaranteed. Use of potable water for washing leafy vegetables reduces the number of bacteria by 0.1–1 log_10_ units, i.e. by 90% at best [25]. Some microorganisms may also adhere to cut surfaces or in stomata and are thus inaccessible to wash water [26].

Studies examining the microbiological quality of RTE leafy vegetables have mainly focused on bacterial pathogens. *Listeria monocytogenes* has usually been isolated at low prevalences in salads, *Salmonella* has occasionally been isolated and STEC and pathogenic *Y. enterocolitica* have generally not been found in investigations of salads [27–30]. However, since very large quantities of leafy vegetables are consumed and most are consumed raw, even minor contamination of salads can pose a risk to public health.

While there have been improvements in surveillance and investigation of foodborne disease outbreaks [31] and improvements in laboratory diagnostics for some pathogens [32], it is still very challenging to confirm leafy vegetables as the source of foodborne disease outbreaks. By the time the outbreak is confirmed, usually 2–4 weeks after the exposure [33], the contaminated leafy vegetables have usually already been consumed or discarded, with nothing left to analyse. In addition, leafy vegetables are often consumed with other foods, in composite foods (such as mixed-ingredient salads and sandwiches) or as garnish [31]. Identifying the contaminated ingredient in such dishes can be difficult, requiring detailed information on the constituent ingredients [31]. Consequently, in most outbreaks associated with leafy vegetables, the association is based on evidence from epidemiological studies. The fact that many of the leafy vegetable products implicated in foodborne disease outbreaks are mixes of varieties with different origins further complicates tracing back possible sources of contamination. In an outbreak of yersiniosis in Norway in 2011 [34], the implicated salad mix contained four different varieties of leafy vegetables, originating from 12 suppliers in two countries. The source of contamination was not identified, but after voluntary withdrawal of the salad mixes by the Norwegian company there were no more reported cases [34].

## Other sources of contamination of mixed-ingredient salads

Foods with leafy vegetables as one of several ingredients, such as mixed-ingredient salads, may be contaminated not only by the leafy vegetables but also by the other ingredients. These ingredients may be raw or processed. For example, meat ingredients may be contaminated during slaughter or in subsequent processing steps. Meat ingredients, for example poultry meat and ham, are generally heat-treated before preparation, but may be contaminated after this treatment, e.g. when diced or sliced. Improper heating or cooling of meat or carbohydrate ingredients may also jeopardise the microbiological safety of these products. When several ingredients are mixed together, cross-contamination may occur. Preparation of mixed-ingredient salads requires human handling, which presents an additional risk of bacterial contamination. Sub-functional cold storage for the final product may also play an important role in determining the microbiological quality of mixed-ingredient salads. When a high-protein food, e.g. cooked meat, is added to leafy vegetables, the final product represents a better substrate for bacterial growth, since denatured protein has high concentrations of accessible nutrients, neutral pH and high water activity [35].

## Bacteriological safety of mixed-ingredient salads

Because of the increased consumption of RTE food, such as mixed-ingredient salads, and the risk they may pose to public health, in 2005 the European Commission (EC) launched a programme to assess the bacteriological safety of mixed-ingredient salads at retail, with presence of *L. monocytogenes* as the primary concern [36]. This resulted in official reports from Ireland and the UK (UK) of *L. monocytogenes* contamination in 2.7% and 4.8%, respectively, of mixed-ingredient salads analysed [2,3] (Table 1). The Irish salad samples were also analysed for *Salmonella*, but this pathogen was not detected. Apart from these official reports, to my knowledge there have been few studies investigating the microbiological quality of mixed-ingredient salads. Results from studies performed in Turkey and Sweden are shown in Table 1, with the highest prevalences of *L. monocytogenes* and *Salmonella* found in the Turkish salads (6.5% and 10.4%, respectively) [5,37]. In the Swedish study, almost 10% of RTE mixed-ingredient salads from retail outlets were found to be contaminated or suspected to be contaminated with foodborne pathogens. *Listeria monocytogenes* was isolated from two out of 141 (1.4%) samples analysed in that study. The other findings included detection of virulence genes present in pathogenic *Y. enterocolitica* and STEC, but these could not be confirmed by culture [5]. *Escherichia coli* as an indicator of faecal contamination was detected in 3.9% of the Turkish mixed-ingredient salads [37], while it was not detected in the Swedish salads [5].

**Table 1.** Prevalence (%) of human pathogens reported in studies of mixed-ingredient salads.

Country, year	Number of salads	*Listeria monocytogenes*	*Salmonella*	STEC	*Campylobacter* spp.	Pathogenic *Yersinia enterocolitica*	Reference
UK, 2005	2686	4.8	–	–	–	–	Little *et al*. [2]
Ireland, 2005	714	2.7	Not detected	–	–	–	Anonymous [3]
Turkey, 2011/2012	154^a^	6.5	10.4	–	–	–	Gurler *et al*. [36]
Sweden, 2012/2013	141	1.4	Not detected	2.1^b^	Not detected	5.0^c^	Söderqvist *et al*.[5]

^a^Including ‘Sezar salad’ (boiled or fried chicken meat, fried bread, lettuce, parsley, tomato, cucumber and boiled corn), tuna fish salad (canned tuna fish meat, parsley, lettuce, tomato, cucumber and boiled corn) and Mediterranean salad (tomato, Turkish white cheese, olive, boiled corn, lettuce, black cabbage, carrot and cucumber)

^b^Detection of *stx1* and/or *stx2* genes

^c^Detection of *ail* gene

There were differences in *L. monocytogenes* prevalence in different categories of salads in the study in the UK, with a higher prevalence in salads containing meat (6%) than in salads containing seafood (3.8%) [2]. However, the highest prevalence, in both the UK and Swedish studies, was found in salads containing salmon, with *L. monocytogenes* present in 10% and 8% of salmon salads analysed, respectively [2,5]. It should be noted that only 12 salads containing salmon were analysed in the Swedish study and that all of these salads contained smoked salmon, which is considered to be an important vehicle of foodborne human listeriosis [38]. For example, *L. monocytogenes* was detected in 12% of smoked salmon samples at retail in Sweden in 2010 [39].

In the UK study of mixed-ingredient salads, *L. monocytogenes* was present more frequently in salads containing chicken and bacon (8.1%) and in salads containing chicken (6.2%) than in salads containing other meat types, e.g. ham (3.0%) and beef (1.7%) [2], which suggests that chicken may be associated with higher levels of contamination. In the Swedish study, *L. monocytogenes* was present in 1% of chicken salads, but was not found in any salad containing ham [5]. Chicken is heat-treated before consumption and this treatment normally eliminates pathogens. However, *L. monocytogenes* may be present in the food processing environment [40] and thus cross-contamination can occur during processes following heat treatment, via for example equipment when slicing or dicing and human handling. However, chicken meat is not known to be an important vehicle of foodborne human listeriosis and *L. monocytogenes* was detected in only 1.6% of the RTE chicken meat analysed in the EU in 2013 [38], which is no higher than the prevalence reported for other common salad ingredients. For example, *L. monocytogenes* has been found in 3% of RTE salads with no meat ingredient [41] and in 1% of samples of ham [39,42]. It appears that many ingredients in a mixed-ingredient salad may be contaminated, and thus the risk of contamination increases with the number of added ingredients in a mixed-ingredient salad.

In the studies in the UK, Ireland and Sweden cited above, most samples were collected from supermarkets (78–94%), while the remainder were collected from e.g. sandwich shops, convenience stores and delicatessens [2,3,5]. The majority of samples in those studies were pre-packed on the production premises (65–93%). In the Swedish study, all salads were packed in plastic bowls with lids and these packs of salads ranged in weight from 310 to 550 g. The recommended storage condition indicated on the label of most salads in that study was maximum 8°C for three days [5]. Based on the contents label of sampled salads in the Swedish study, the most common leafy vegetable ingredient was iceberg lettuce. Among other ingredients, tomato was the most common, followed by carrot and pasta. In the studies in the UK, Ireland and Sweden, the most common meat ingredient in the salads analysed was chicken.

## Levels of Listeria monocytogenes and EU microbiological criteria

The microbiological criteria in EC Regulation No. 2073/2005 [43] for *L. monocytogenes* set a limit of ≤100 CFU/g for RTE products during the whole shelf-life, which is considered to pose a negligible risk to a healthy population [38,43]. If this criterion is not met, the product is deemed not acceptable for consumption. In the studies in the UK [2] and Ireland [3], only 0.1–0.3% of samples contained levels of *L. monocytogenes* exceeding 100 CFU/g, with the levels in those samples ranging from 170 to 1200 CFU/g. Thus, the vast majority of samples in those studies complied with the legal food safety criteria for *L. monocytogenes*. The levels of *L. monocytogenes* in positive samples were not reported in the Swedish or Turkish studies [5,37].

Food products can be considered to support microbial growth when the numbers of *L. monocytogenes* increase by more than 0.5 log CFU/g during shelf-life [44]. However, when interpreting the EU microbiological criteria [43], RTE products with a shelf-life of less than five days, such as mixed-ingredient salads, are classified as RTE foods unable to support growth of *L. monocytogenes*. For RTE foods able to support growth, *L. monocytogenes* must be absent before the product leaves the food business operator [43], while for RTE food unable to support growth no testing is needed to show absence at this point. For both categories, however, the level of *L. monocytogenes* should be less than 100 CFU/g during its shelf-life.

## Storage temperature

The recommended storage temperature in retail differs between countries, but all food business operators in the EU must ensure that their food products are safe and that they meet food safety criteria throughout their shelf-life under reasonably foreseeable conditions of distribution, storage and use [43]. For example, in the Nordic countries the recommended storage temperature varies from 3 to 8°C, with the lowest temperature applying in Finland (cold smoked salmon) and the highest temperature in Sweden [45]. For RTE salad with chicken, the maximum recommended temperature is 4 °C in Norway, 5 °C in Denmark and 6°C in Finland [45]. In Sweden, maximum 8°C is usually indicated on the pack label of RTE salads with chicken and these products often have a shelf-life of three days [5].

Regardless of the recommended storage temperature, many studies report temperature abuse in both retail [46] and domestic refrigerators [47,48]. In the UK study, 93.2% of the salad samples for which temperature was recorded were displayed at or below 8°C, while 5.9% were stored at 9–24°C [2]. In the Irish study, 23.8% of salads were stored at temperatures >5 °C, while 6.9% were stored at >8°C and 15.9°C was the highest storage temperature recorded [3].

## Growth of human pathogens in mixed-ingredient salads

To my knowledge, there have been few studies investigating growth of human bacterial pathogens in mixed-ingredient salads. Results from experiments by Bovo et al. [4] showed that populations of *S. enterica* increased significantly when romaine lettuce was incubated in contact with cooked chicken at 14°C, i.e. temperature abuse, but no growth was observed when romaine lettuce was incubated alone. Since the contact area between lettuce surfaces and added ingredients in a commercial mixed-ingredient salad may be limited, because of non-uniform distribution of ingredients (Figure 1), experiments including commercial mixed-ingredient salads containing romaine salad, shredded Cheddar cheese and cooked chicken were also included [4]. In these packages, one-third of romaine lettuce was replaced with lettuce inoculated with *S. enterica* and it was found that populations increased by 4 log CFU/g during storage for three days (length of shelf-life) at 14°C, but no growth was observed at 6°C. *Salmonella enterica* did not grow at 14°C in salads with plain romaine lettuce or on lettuce in contact with Cheddar cheese, despite cheese being a rich source of growth substrates. It was speculated that low pH and effects of lactic acid resulting from contact with Cheddar cheese may have restricted growth of *S. enterica* [4].

In Sweden, 8°C is the recommended maximum storage temperature for mixed-ingredient salads. However, in inoculation trials by Söderqvist et al. [49] significant growth of *L. monocytogenes* occurred in baby spinach mixed with chicken meat at this temperature during shelf-life, with numbers increasing by approximately 1 log CFU/g during three days. Since this exceeded 0.5 log CFU/g, which is the maximum permissible growth during shelf-life for a food product that does not support growth of *L. monocytogenes* [44], it indicated that mixed-ingredient salads may support growth of this pathogen. When baby spinach mixed with chicken meat was stored at 15°C, *L. monocytogenes* increased from 50–100 CFU/g to approximately 7.0 log CFU/g during a shelf-life of three days. In comparison, in plain baby spinach growth of *L. monocytogenes* was not observed at 8°C, while limited growth was observed at 15°C.

To explore the food safety implications of these findings, bacterial numbers were translated into risk of infection by modelling the dose-response curves and exposures per portion mixed-ingredient salad (300 g) [49]. The risk of listeriosis was estimated to be 16-fold higher on consuming a mixed-ingredient salad stored at 8°C at the end of shelf-life, or 200 000-fold higher when the salad was stored at 15°C, compared with consumption on the day of inoculation [49]. Hence, preventing temperature abuse during storage is of critical importance in mitigating the risk of foodborne listeriosis from these mixed-ingredient salads.

In the inoculation trials [49], growth of pathogenic *Y. enterocolitica* and *E. coli* O157:H7 gfp+ was also tested. Mixing RTE baby spinach with chicken meat strongly influenced growth of inoculated strains during storage under temperature abuse (15°C), with populations of *Y. enterocolitica* and *E. coli* O157:H7 gfp+ increasing from 50–100 CFU/g at the day of inoculation to mean concentrations of 4.0 and 5.6 log CFU/g, respectively, after three days. No growth was observed in baby spinach mixed with chicken stored at 8°C. *Escherichia coli* O157:H7 gfp+ did not grow in plain baby spinach, regardless of whether it was stored at 8 or 15°C, but pathogenic *Y. enterocolitica* grew in plain baby spinach stored at 15°C, reaching approximately 3.3 log CFU/g after three days, but not at 8°C.

## Conclusions

Based on different studies, *L. monocytogenes* can be expected to be present in a few percent of mixed-ingredient salads at retail. Any ingredient in a mixed-ingredient salad may be contaminated by human bacterial pathogens, and thus the risk of contamination increases with the number of added ingredients in a mixed-ingredient salad. Only a few studies have investigated growth of pathogens in mixed-ingredient salads. However, it appears that mixing of RTE leafy vegetables with cooked chicken strongly influences growth of *Salmonella, L. monocytogenes*, pathogenic *Y. enterocolitica* and *E. coli* O157:H7, with rapid growth during storage under temperature abuse. Preventing temperature abuse during storage is therefore of critical importance in mitigating the risk of foodborne disease from mixed-ingredient salads. Manufacturers preparing mixed-ingredient salads should aim to eliminate contamination by pathogens wherever possible and check the ingredients and final mixed-ingredient salad before entering the market.
